# Genome-wide linkage and association mapping identified a novel candidate gene, *TaGLK-A1*, underlying fructan content in wheat grain

**DOI:** 10.1007/s00122-026-05345-z

**Published:** 2026-08-19

**Authors:** Zhankui Zeng, Yue Zhao, Junge Bi, Qunxiang Yan, Wei Zhang, Feng Chen, Chunping Wang

**Affiliations:** 1 College of Agronomy, Henan University of Science and Technology, Luoyang, 471000 Henan China; 2 Shandong Provincial Postdoctoral Innovation and Practice Base, Shandong Xinfeng Seed Industry Co., Ltd,, Liaocheng, 252000 Shandong China; 3 Luoyang Agricultural Technology Extension Service Center, Luoyang, 471000 Henan China; 4 College of Agronomy/National Key Laboratory of Wheat and Maize Crop Science/Collaborative Innovation Center of Henan Grain Crops, Henan Agricultural University, Zhengzhou, 450046 Henan China

## Abstract

**Key message:**

A nuclear-localized G2-like transcription factor, *TaGLK-A1* (*TraesCS7A02G539600*), was identified as a strong candidate gene for grain fructan content in wheat using GWAS, linkage mapping and transcriptome analysis.

**Abstract:**

Fructans, a type of natural polysaccharides or oligosaccharides polymerized from fructose molecules, play important physiological roles in crops and confer great health benefits to humans. In this study, we detected 78 stable loci associated with fructan content across all 21 wheat chromosomes across three environments and in the BLUE, explaining 4.4–10.0% of the phenotypic variance by a genome-wide association study (GWAS) using 243 wheat cultivars and advanced lines genotyped with the Axiom wheat 660 K single-nucleotide polymorphism (SNP) array. Among these, a significant locus of fructan content was localized in a 4.02 Mb interval (713.87–717.89 Mb) on chromosome 7AL, designated *QGfc.hkd-7A*. *QGfc.hkd-7A* was validated in 164 recombinant inbred lines (RILs) from a cross between Avocet and Chilero in five environments using diversity array technology (DArT), which was tightly linked to DArT markers *SNP2260399*, *3575400*, *SNP3028492*, *SNP100415949*, *3533474* and *100091413*, accounting for 16.4–24.6% of the total phenotypic variance in the RIL population. Favorable allele of *QGfc.hkd-7A* significantly increased grain fructan content by 10.5% (*P* < 0.01) compared with lines carrying unfavorable alleles. To further validate *QGfc.hkd-7A*, we generated differential gene expression profiles using RNA-seq for high-fructan (AC6012) and low-fructan (AC6361) lines from the Avocet/Chilero population. *TaGLK-A1* (*TraesCS7A02G539600*), a differential expressed gene (DEG), was subsequently cloned and sequenced from both genotypes. Sequence comparison of the full-length *TaGLK-A1* cDNA (1545 bp) revealed a single SNP at position 109 causing a proline (CCA) to threonine substitution between Avocet and Chilero. Subcellular localization analysis confirmed that the G2-like transcription factor encoded by *TaGLK-A1* localizes to the nucleus. Transactivation assays revealed that it lacks intrinsic transcriptional activation activity, suggesting it may function as a transcriptional repressor or require cofactors for regulatory function. These results provide a novel and strong candidate gene for fructan content and a forward genetics strategy for gene discovery in molecular breeding.

**Supplementary Information:**

The online version contains supplementary material available at https://doi.org/10.1007/s00122-026-05345-z.

## Introduction

Wheat (*Triticum aestivum* L.) provides an important source of dietary fiber for humans (Liu et al. 2023; Zhang et al. 2022). Fructans, one of the major components of the dietary fiber, are beneficial for human intestinal health because they promote the proliferation of beneficial bacteria while preventing the growth of detrimental bacteria (Ford et al. 2014). Dietary fructans also play an active role in regulating the metabolism of serum glucose and lipids (Dapa et al. 2022; Dehghan et al. 2014; Desai et al. 2016). Nevertheless, as FODMAPs (fermentable oligosaccharides, disaccharides, monosaccharides and polyols), fructans may trigger gastrointestinal symptoms in sensitive individuals, including those with irritable bowel syndrome (IBS) (Gibson et al. 2014). In addition, fructans rather than gluten have been implicated in some cases of self-reported non-celiac gluten sensitivity (NCGS) (Biesiekierski et al. 2013; Skodje et al. 2018). Beyond their dietary significance, fructans are important factors in many physiological processes in wheat development, where they serve as osmotic regulators to conserve carbohydrates and affect grain yield (Ribeiro et al. 2022).

Fructans are carbohydrate polymers consisting of a sucrose unit linked to one or more fructose units by glycosidic bonds (Ávila-Fernández et al. 2023; Roberfroid and Delzenne 1998) and are classified into five main classes (inulin, levan/phlein, graminan, neoinulin and neolevan) according to the degree of polymerization (DP) and types of glycosidic bonds (Harrison et al. 2012; Van den Ende 2013). In wheat, fructans include branched graminan- and linear levan-type forms (Al-Sheikh Ahmed et al. 2020) with a low DP, ranging from 4 to 10 (Wardlaw and Willenbrink 1994). Their metabolism is regulated by fructosyltransferases (FSTs) and fructan exohydrolases (FEHs) (Veenstra et al. 2017; Vijn and Smeekens 1999).

Fructans are the major components of water-soluble carbohydrates (WSCs) in stems (McIntyre et al. 2012), acting as osmoregulators and playing important roles in defense against multiple stresses (Ehdaie et al. 2006; Xue et al. 2008; Zhang et al. 2009). Several studies have shown that the remobilization of fructans is extremely important under drought conditions (Joudi et al. 2012; Kühbauch and Thome 1989), in which reserved carbohydrates in stems are remobilized from vegetative tissues to developing wheat grains (Palta et al. 1994; Yang et al. 2016, 2020). Fructans also play an osmoregulatory role under cold, salt, waterlogging and heavy metal stress (Frossard et al. 1989; Gibson 2005; Kerepesi and Galiba 2000; Ohtake et al. 2006). In addition to the regulation of metabolic and physiological processes, fructans are also important for wheat grain development. The fructan content in wheat grains varies across three development stages during grain filling and maturation, viz. peaking at up to 30% of dry weight (5 days post-anthesis), decreasing during the rapid starch accumulation phase (2–3 weeks post-anthesis) and stabilizing at 0.7–2.9% of total dry weight in mature grains (Pepler et al. 2006; Shewry et al. 2012; Paradiso et al. 2008; Gara et al. 2003; Cimini et al. 2015; Verspreet et al. 2013; Huynh et al. 2008a).

With the rapid development of molecular biotechnology, many reports on quantitative trait locus (QTL) mapping and cloning of genes involved in fructan metabolism in plants have been published (Bian et al. 2018; He and Huang 2013; Kawakami and Yoshida 2002; Márquez-López et al. 2022). QTL mapping for fructan content in wheat grain was first reported by Huynh et al. (2008b), who detected eight QTLs on chromosomes 2B, 2D, 3B, 5A, 6D and 7A using a doubled haploid (DH) population, in which *QGfc.aww-6D.2* and *QGfc.aww-7A.1* had the greatest effects, accounting for 17% and 27% of the total phenotypic variance, respectively. The fructan synthesis gene (*6-SFT*) was isolated from common wheat aiming at revealing the mechanisms underlying resistance to adverse environmental conditions (Gao et al. 2009). This gene was mapped to chromosome 4A using a recombinant inbred line (RIL) population (Yue et al. 2011). Eleven QTLs for fructan content in wheat grain were mapped to chromosomes 1B, 2A, 2B, 2D, 3D, 4B, 6B, 6D, 7A and 7B, accounting for 4.1–27.4% of the phenotypic variance (Zeng et al. 2020). In another study, 82 additive QTLs explaining 0.02–16.1% of the phenotypic variance in the accumulation and transport of fructan in wheat stems and leaves were detected on all wheat chromosomes except 1A and 5D. The epistatic QTLs explaining 0.8–14.1% of the phenotypic variance in fructan transport-related traits were mapped to 21 wheat chromosomes (Lv 2019). In addition, the fructan 1-exohydrolase (*1-FEH*) gene was cloned, and KASP markers were developed for wheat breeding (Fu 2020).

High fructan content in wheat grain is important for increasing both fructan intake by humans and the resistance of wheat seedlings to adverse stresses (Kerepesi and Galiba 2000). The objectives of this study were to 1) discover new marker–trait associations for fructan content in wheat grain using GWAS, 2) validate the important QTL through linkage mapping, 3) analyze differentially expressed genes via transcriptomic approaches and identify candidate genes for the new QTL, and 4) clone this candidate gene and perform regulatory network analysis to elucidate its potential involvement in fructan metabolism in wheat grain.

## Materials and methods

### Plant materials

A diverse panel of 243 wheat cultivars and advanced lines (designated “CH population”) was used for GWAS. These lines were released or developed between 2014 and 2020 in the Huanghuai wheat region of China and represent recent widely used wheat breeding resources. This population was previously used for studies on water-extractable arabinoxylan, Fusarium crown rot and black point (Li et al. 2024; Lv et al. 2020; Yang et al. 2019).

A biparental RIL population with 164 F_6_ lines (designated “AC population”) derived from a cross between Avocet and Chilero via the single-seed descent method was obtained from the International Maize and Wheat Improvement Center (CIMMYT) (Basnet et al. 2014).

### Field trials

The CH population was planted at the experimental station of Mengjin County (34°83'N, 112°58'E) during the 2019–2020 and 2020–2021 cropping seasons and at the farm of Henan University of Science and Technology (34°35'N, 112°25'E) during the 2020–2021 cropping season.

The AC population was planted at the experimental station of Luoning County (34°42'N, 111°66'E) in 2019–2020, at the experimental station of Mengjin County during the 2019–2020 and 2020–2021 cropping seasons, and at the farm of Henan University of Science and Technology in 2019–2020 and 2020–2021.

Field trials were performed in randomized complete blocks with three replications. Each plot was 1 m long, comprising 10 rows with a row spacing of 25 cm. Field management was performed in accordance with local wheat cropping practices. No major pest- or disease-related problems occurred during the field trials.

### Phenotypic evaluation

After harvest, the grains of each wheat cultivar were dried to approximately 11% moisture content. Wheat grains (5 g) were selected from a well-mixed sample, and each sample was subsequently milled via a tissue grinding apparatus (MM400, RETSCH) for 30 s to obtain whole wheat flour. To determine the fructan content, the whole wheat flour was weighed, and 0.1 g was transferred to a 10-mL centrifuge tube. Then, 10 mL of distilled water was added to the centrifuge tube, and the sample was incubated in a water bath at 80℃ for 60 min. The sample was centrifuged at 6000 r/min for 5 min, and 0.5 mL of the supernatant was mixed with 0.5 mL of distilled water in a test tube with a glass stopper. A total of 1.5 mL of 0.5 g/L resorcinol solution and 1.5 mL of 0.216 g/L ferric ammonium sulfate–hydrochloric acid solution were added to the tube, followed by incubation in a water bath at 80℃ for 50 min. The sample was allowed to cool to room temperature, followed by absorbance measurement at 473 nm. Three independent measurements of fructan content were taken on each sample. The best linear unbiased estimate (BLUE) was calculated and used in the subsequent statistical analysis.

### Phenotypic data analysis

Statistical analyses, including descriptive statistical variable analysis, correlation analysis and variance analysis, were performed using SPSS 19.0 software. Graphs were plotted via Origin software, version 2022b, and the “ggplot2” package in R software. The BLUE for each wheat genotype was calculated via the analysis of variance (ANOVA) function of QTL IciMapping software, version 4.2 (http://www.isbreeding.net). The ANOVA function was used to calculate broad-sense heritability (*H*^*2*^) via the following equation (Yan et al. 2022):

$${\mathrm{H}}^{2} = \frac{{\sigma_{g}^{2} }}{{\sigma_{g}^{2} + \sigma_{ge}^{2} + \frac{{\sigma_{\varepsilon }^{2} }}{n}}}$$

where ${\sigma}_{g}^{2}$, ${\sigma}_{ge}^{2}$, ${\sigma}_{\varepsilon }^{2}$ and n are the genetic variance, the interaction variance between genotype and environment, the residual error variance and the number of environments, respectively.

### GWAS

The CH population was genotyped via the wheat 660 K SNP assay, as previously described (Lv et al. 2020; Yang et al. 2019). Details about genotyping, quality control, principal component analysis (PCA) and population structure analysis can be found in Yang et al. (2019). In brief, quality control evaluation of the genotypic data was performed via PLINK software with the thresholds maf 0.02 and geno 0.1 (http://zzz.bwh.harvard.edu/plink/tutorial.shtml) (Purcell et al. 2007). After SNP filtering, 395,783 SNP markers were retained for GWAS.

The combined mixed linear model (PCA + K) was constructed via the GAPIT program of R software (http://www.maizegenetics.net/gapit) (Zhang et al. 2010), and the variance–covariance kinship matrix (K) was generated according to the VanRaden method (VanRaden 2008). To identify additional markers potentially linked to fructan content, a threshold of -log10 (*P*) = 3 was used to determine statistically significant associations between markers and traits. The significant SNP markers detected in the same linkage disequilibrium (LD) block were merged into a single QTL. The LD values in the A, B and D genomes were 3 Mb, 10 Mb and 3 Mb, respectively (Lv et al. 2020; Yang et al. 2019). A QTL was considered stable if it was detected in at least two environments (Guan et al. 2022).

### QTL analysis

Whole-genome genotyping data of 23,526 diversity array technology (DArT) markers on both parents and 164 RILs were generated via the DArT genotyping platform. After quality control, 3,290 DArT markers were used for constructing the genetic map via the QTL IciMapping, version 4.2 (https://isbreeding.caas.cn/rj/index.htm).

QTL mapping was carried out via the inclusive composite interval mapping (ICIM) method in QTL IciMapping, version 4.2. The threshold for the detection of significant QTLs was a fixed LOD value of 3.0.

QTL and gene nomenclature followed the international guidelines for wheat (Boden et al. 2023). The QTL for grain fructan content was designated *QGfc.hkd-7A*, where “Gfc” follows the trait abbreviation of Huynh et al. (2008a, b) and “hkd” is our registered laboratory code. The strong candidate gene, *TraesCS7A02G539600*, identified within the *QGfc.hkd-7A* interval was named *TaGLK-A1*.

### Transcriptome experiment

Lines with high fructan content (AC6012) and low fructan content (AC6361) from the AC population were used for transcriptome analysis. These two lines exhibited significantly different wheat grain fructan contents across five environments and showed clear genotypic differentiation at the closely linked markers in the *QGfc.hkd-7A* locus region: AC6012 displayed the paternal genotype at all closely linked markers, while AC6361 showed the maternal genotype at the same closely linked markers. The developing grain samples at 15 d post-anthesis were collected from AC6012 and AC6361 and frozen in liquid nitrogen for transcriptome analysis, with three biological replicates. All samples were sent to Biomarker Technologies (Beijing Biomarker Biotechnology Co., Ltd., Beijing, China) for transcriptome sequencing and analysis.

### Identification of putative candidate genes

The physical intervals of the identified QTLs were determined via BLAST searches against the reference sequence of Chinese Spring (http://plants.ensembl.org/index.html) using the marker sequences as queries. Candidate genes within the physical intervals of validated loci were extracted from WheatGmap (Zhang et al. 2021; Zhao et al. 2022).

## Gene cloning

### Amplification of target fragments

DNA amplification was performed using KOD HiFi DNA polymerase to ensure high efficiency and accuracy. A reaction mixture (30 μL) was prepared, consisting of 2 × KOD buffer (15.0 μL), dNTPs (6 nmol, 0.6 μL of 10 mM stock), DNA template (100 ng, 1.0 μL of 100 ng/μL stock), forward primer (Primer F, 0.2 μM final concentration, 0.6 μL of 10 μM stock), reverse primer (Primer R, 0.2 μM final concentration, 0.6 μL of 10 μM stock), KOD FX enzyme (0.8 U, 0.8 μL of 1 U/μL stock) and ddH_2_O (11.4 μL). The amplification program included an initial denaturation at 94 °C for 2 min, followed by 36 cycles of denaturation at 98 °C for 20 s, annealing at 58 °C for 30 s and extension at 68 °C for 2 min, with a final extension at 68 °C for 10 min.

PCR products were separated via 1% agarose gel electrophoresis under 120 V for 20–30 min. The gel was visualized using the Gel Doc^TM^ XR + system (Bio-Rad). The desired band corresponding to the target size was excised from the gel for purification.

### Recovery of purified fragments

The GeneJET Gel Extraction Kit (Thermo-Scientific) was employed for DNA recovery. Under UV light, the gel slice containing the target fragment was excised and placed in a pre-sterilized 1.5-mL centrifuge tube with an equal weight of binding buffer. The mixture was incubated at 60 °C for 10 min, followed by centrifugation to remove supernatant. Wash buffer was added twice, followed by centrifugation again to eliminate impurities. The DNA pellet was resuspended in elution buffer and stored at 4 °C.

### Cloning and sequencing

Purified PCR products were cloned into competent E. coli cells (Trans1-T1 strain) via a ligation reaction. Following transformation, cells were incubated on ice for 30 min, subjected to heat shock at 42 °C for 30 s and cultured in SOC medium at 37 °C with shaking (200 rpm) for 2 h. Single colonies were selected on LB agar containing appropriate antibiotics and cultured overnight. Positive clones were sequenced using Sanger sequencing by a commercial provider.

### Subcellular localization

The constructs 35S:TaGLK-A1-GFP, 35S:BZR1-BFP (nuclear marker) (Zheng et al. 2025) and 35S:SPER-mKATE (endoplasmic reticulum marker) (Nelson et al. 2007) were individually introduced into *Agrobacterium tumefaciens* strain GV3101. The three transformed Agrobacterium suspensions were mixed at a 1:1:1 ratio prior to infiltration and infiltrated into tobacco (*Nicotiana benthamiana*) leaves. Tobacco plants were grown in a greenhouse at 25 °C with a 16-h light/8-h dark photoperiod in 2025. Fluorescence signals were observed 2 d post-infiltration using a Nikon C2 confocal laser scanning microscope.

### Transactivation activity assay

The coding sequence of *TaGLK-A1* was inserted into the pGreenII-62sk-GAL4 vector to create the effector construct pGreenII-62sk-GAL4-TaGLK-A1. This construct was co-expressed with the reporter construct pGreenII 0800-GAL4-TATA in Nicotiana tabacum leaves via Agrobacterium-mediated transient transformation, as described by Hellens et al. (2005). The constructs pGreenII-62sk-GAL4-VP16 and pGreenII-62sk-GAL4 were utilized as positive and negative controls, respectively. Luciferase activity was quantified 48 h post-infiltration using the Dual-Luciferase Reporter Assay System (Vazyme), with Renilla luciferase serving as an internal control. The data are presented as means ± standard deviation from three independent biological replicates.

## Results

### Phenotypic variation and correlation analysis of fructan contents

**Fig.1:**
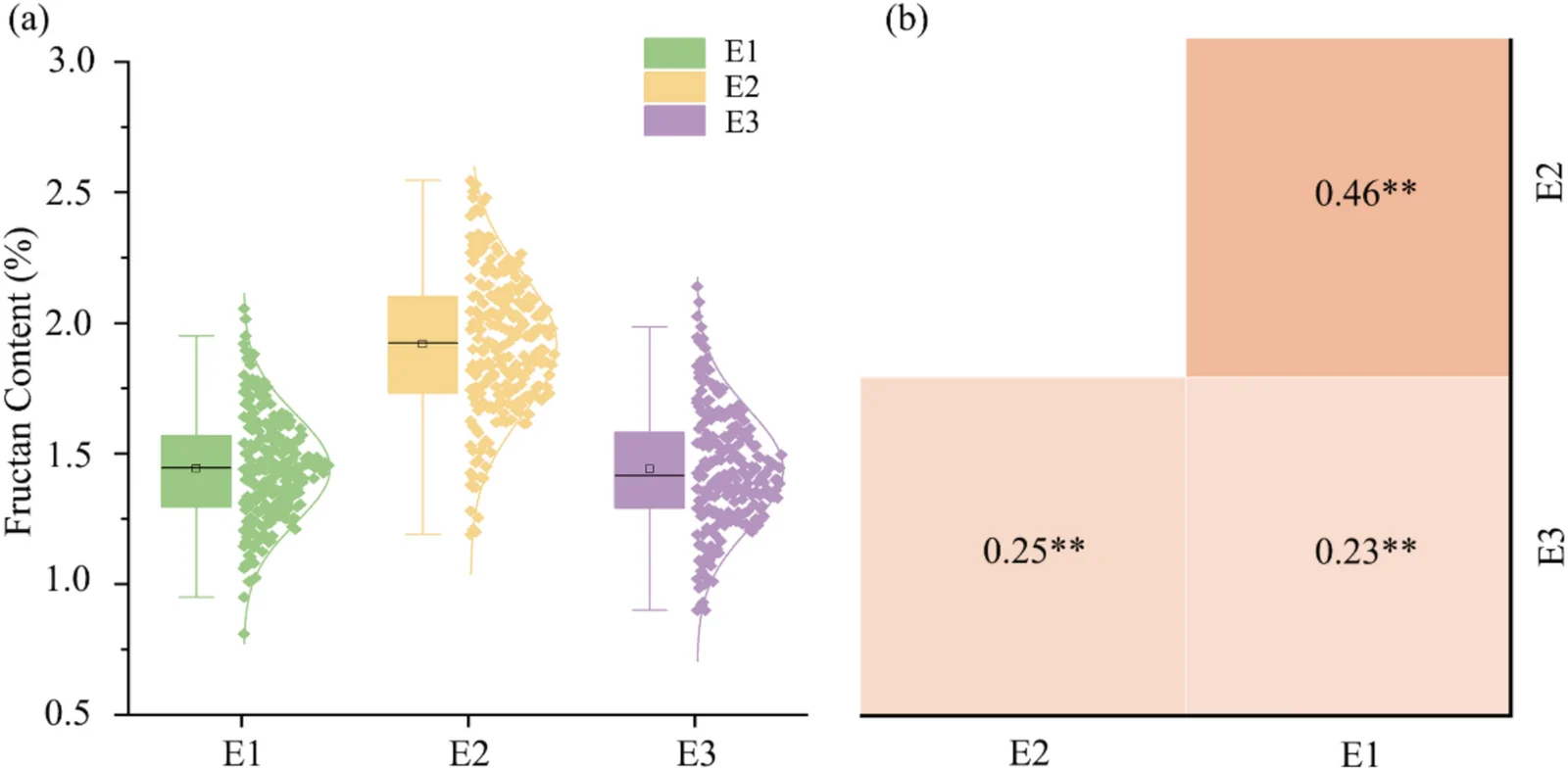
Frequency distribution histogram and correlation analysis of fructan content in the CH population. **a** Frequency distribution histogram of fructan content in the CH population. **b** Heatmap of correlation analysis in the CH population. In the CH population, E1, E2 and E3 represent the data from Mengjin County in 2019–2020, Mengjin County in 2020–2021 and the farm of Henan University of Science and Technology in 2020–2021, respectively. The same below

The fructan content of CH and AC populations was measured in three and five environments, respectively. In the CH population, fructan content in wheat grains ranged from 0.8 to 2.6% and the CV ranged from 11.3 to 15.7% across three environments. Continuous variation was also observed across environments (Fig. 1a), consistent with fructan content being a typical quantitative trait controlled by multiple genes. ANOVA of fructan content across multiple environments revealed that the effects of genotype, environment and the genotype–environment interaction were highly significant (*P* < 0.001). The broad-sense heritability was 0.69, suggesting that the fructan content was mainly controlled by genetic factors. Correlation analysis revealed significant correlations (*P* < 0.01) in fructan content across environments, indicating the relative stability of this trait across three environments (Fig. 1b).

**Fig.2:**
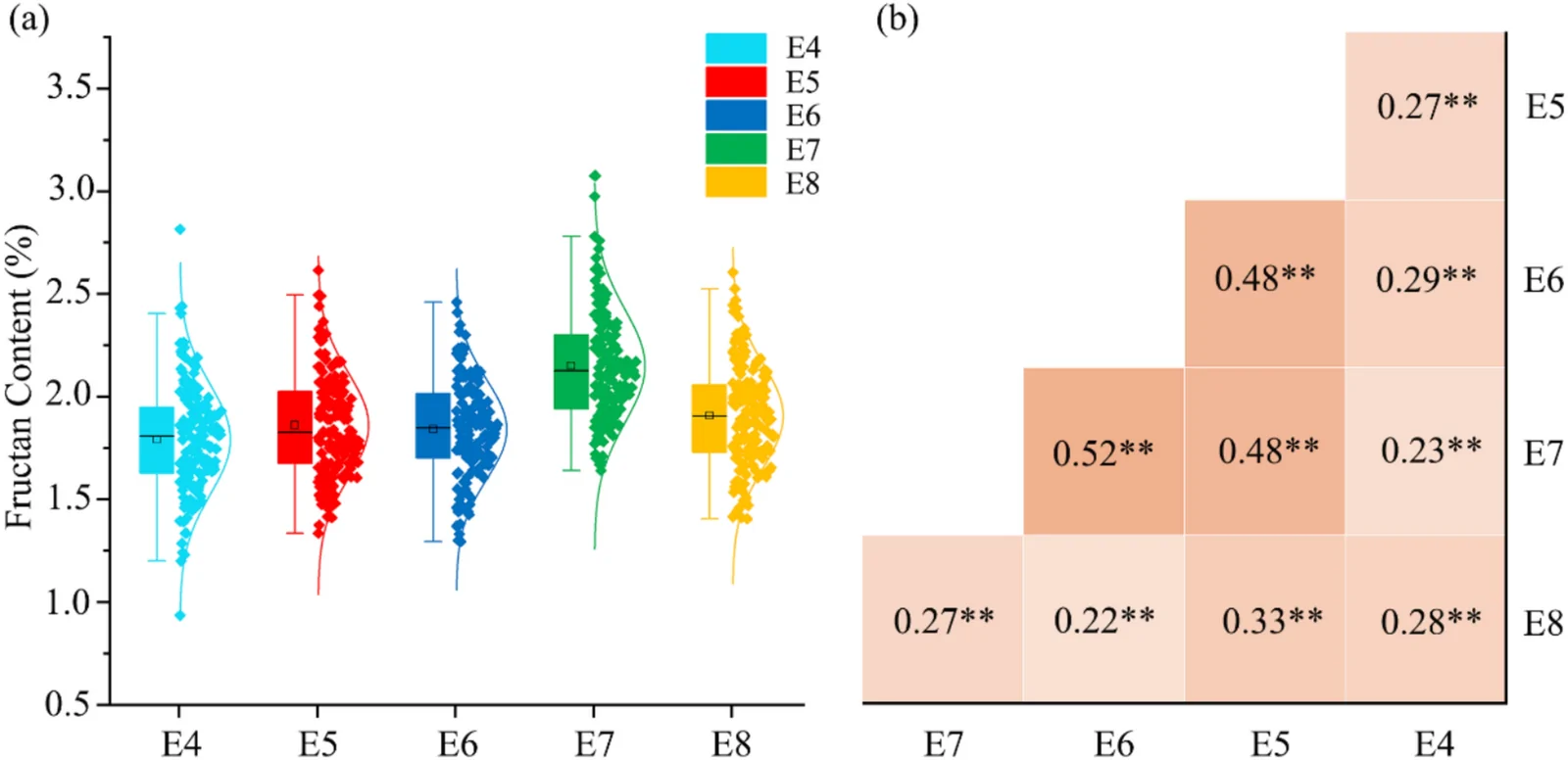
Frequency distribution histogram and correlation analysis of fructan content in the AC population. **a** Frequency distribution histogram of fructan content in the AC population. **b** Heatmap of correlation analysis in the AC population

Significant differences in fructan content were observed between Avocet and Chilero, with Chilero demonstrating higher fructan content (*P* < 0.01) (Table 1). A substantial range of phenotypic variation was evident across all environments, with fructan content ranging from 1.2 to 3.1%. The frequency distribution of fructan content approximated a normal distribution across all environments in the AC population, consistent with a multi-genic nature for this trait (Fig. 2a). ANOVA indicated that fructan content was significantly affected by genotype (G), environment (E) and G × E interaction (*P* < 0.01). The broad-sense heritability (*H*^*2*^) was 0.77 (Table 1). The fructan content showed significant correlations across environments (Fig. 2b).

**Table 1:** Descriptive statistics and ANOVA of fructan contents in the CH and AC populations

Population	Environment	Avocet (%)	Chilero (%)	$\overline{{\boldsymbol{\chi}} }$±${{\boldsymbol{S}}}_{\overline{{\boldsymbol{X}}} }$	Range (%)	CV (%)	ANOVA			H2
							Genotype	Environment	G × E	
CH population	E1	–	–	1.4 ± 0.2	0.8–2.1	14.3	51.2^**^	10,403.4^**^	22.4	0.69
	E2	–	–	1.9 ± 0.3	1.2–2.6	14.1			**	
	E3	–	–	1.4 ± 0.2	0.9–2.1	15.7				
	BLUE-CH	–	–	1.5 ± 0.2	1.1–1.9	11.3				
AC population	E4	1.2	1.6**	1.8 ± 0.3	0.9–2.8	14.7	78.2^**^	1556.6^**^	22.7	0.77
	E5	1.6	1.9**	1.8 ± 0.2	1.3–2.5	13.1			**	
	E6	1.4	1.6**	1.9 ± 0.3	1.3–2.6	13.6				
	E7	1.8	2.1**	2.1 ± 0.3	1.6–3.1	12.4				
	E8	1.2	1.7**	1.9 ± 0.3	1.4–2.6	13.2				
	BLUE-AC	1.4	1.8**	1.9 ± 0.2	1.4–2.5	9.4				

$\overline{\mathrm{X} }$, Mean value; ${S}_{\overline{X} }$, Standard error; G × E, genotype-by-environment interaction; “-” indicates no data. “**” indicates significance at* P* < 0.01. In the AC population, E4, E5, E6, E7 and E8 represent the data from Luoning County in 2019–2020, Mengjin County in 2019–2020, the farm of Henan University of Science and Technology in 2019–2020, Mengjin County in 2020–2021 and the farm of Henan University of Science and Technology in 2020–2021, respectively. BLUE-CH and BLUE-AC represent the BLUE values of fructan content in the CH population and AC population, respectively. The same below

### Significant loci associated with fructan content revealed by GWAS

**Fig. 3:**
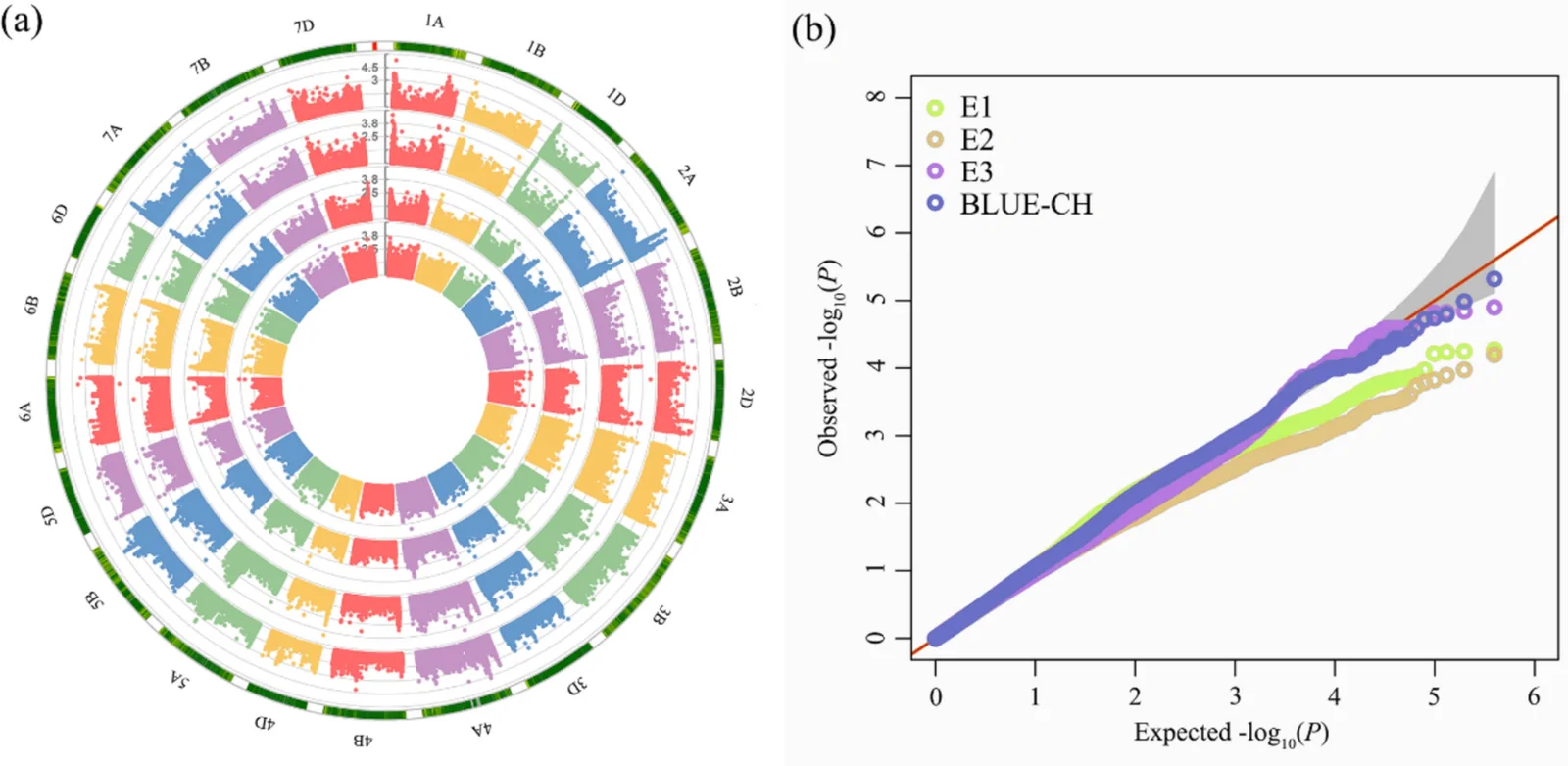
Genome-wide association study (GWAS) results for fructan content. **a** Manhattan plot showing − log₁₀(*P*-values) of markers across all 21 wheat chromosomes. For each chromosome, the left segment represents the short arm and the right segment represents the long arm, separated by the centromere position. The horizontal dashed line indicates the significance threshold (*P* < 0.001, − log₁₀(*P*) = 3.0). **b** Quantile–quantile (QQ) plot for fructan content

Using a mixed linear model (PCA + K), significant markers were detected on all 21 wheat chromosomes (Fig. 3). Fifty-six stable loci were detected in two environments and 22 in three or more environments, explaining 4.4–10.0% of the phenotypic variance. Among these, one locus significantly associated with fructan content was mapped to a 4.02 Mb interval (713.87 Mb-717.89 Mb) on the long arm of chromosome 7A, designated *QGfc.hkd-7A*, and its physical interval (713.87 Mb-717.89 Mb) contains 47 high-confidence genes.

### QTL mapping of fructan content in the AC population

**Fig. 4:**
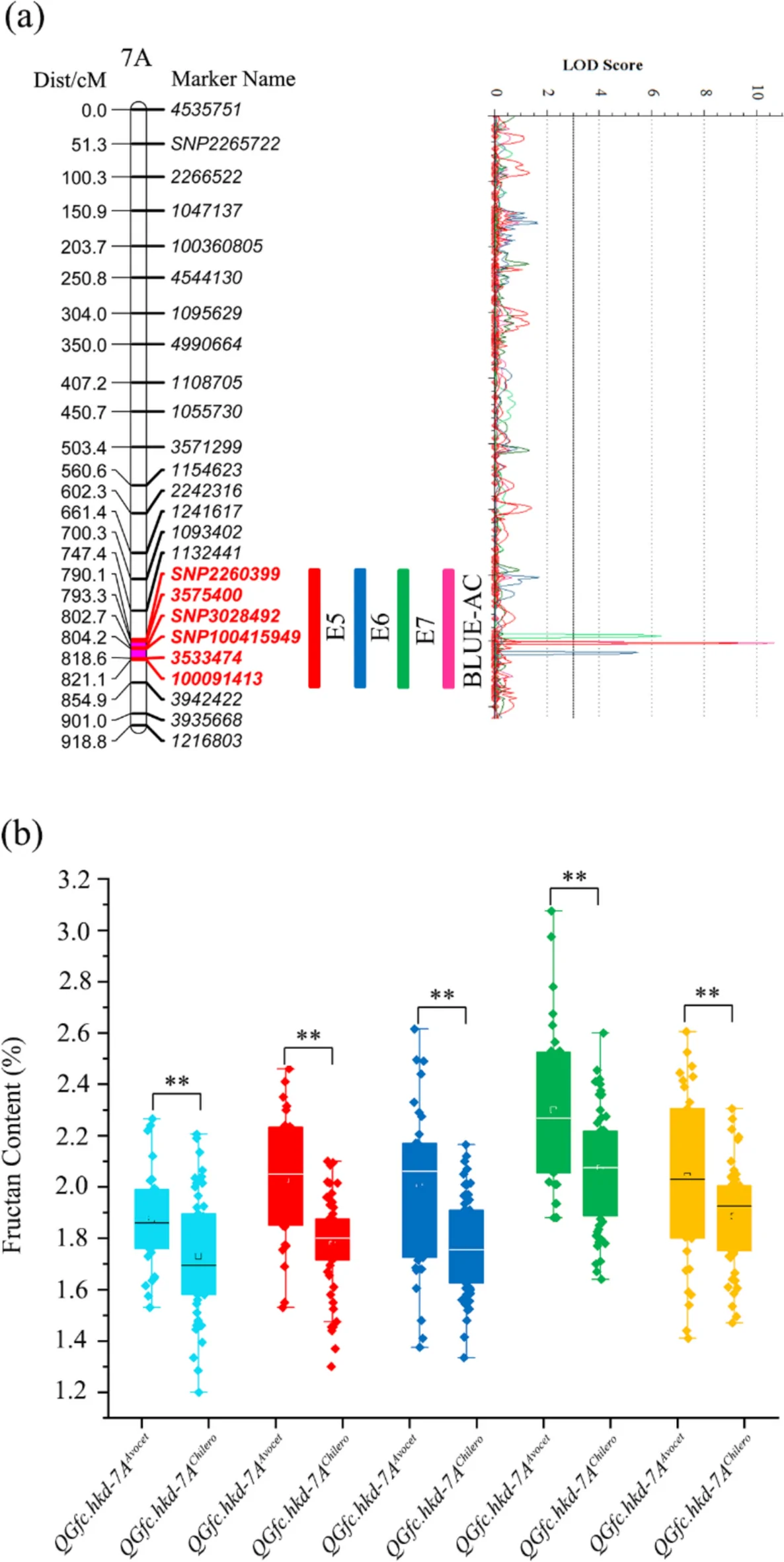
Genetic linkage map and analysis of allelic effects of *QGfc.hkd-7A* in the AC population. **a** Genetic linkage map of *QGfc.hkd-7A*. **b** Analysis of allele effects of *QGfc.hkd-7A* for fructan content in the AC population across five environments

QTLs for wheat grain fructan content were identified on chromosomes 1B, 1D, 2A, 2D, 3A, 6B, 7A and 7B. Most importantly, the *QGfc.hkd-7A* identified in GWAS was also successfully detected through linkage analysis in the AC population (Fig. 4a). This QTL was tightly linked with the DArT markers *SNP2260399*, *3575400*, *SNP3028492*, *SNP100415949*, *3533474* and *100091413*, explaining 16.4–24.6% of the phenotypic variance.

Allelic effect analysis revealed that lines carrying the favorable allele at the *QGfc.hkd-7A* locus had significantly higher fructan content than those carrying the unfavorable allele in all environments (*P* < 0.01), with an average 10.5% increase in grain fructan content (Fig. 4b).

### Transcriptome analysis of *QGfc.hkd-7A* for fructan content in wheat grain

**Fig. 5:**
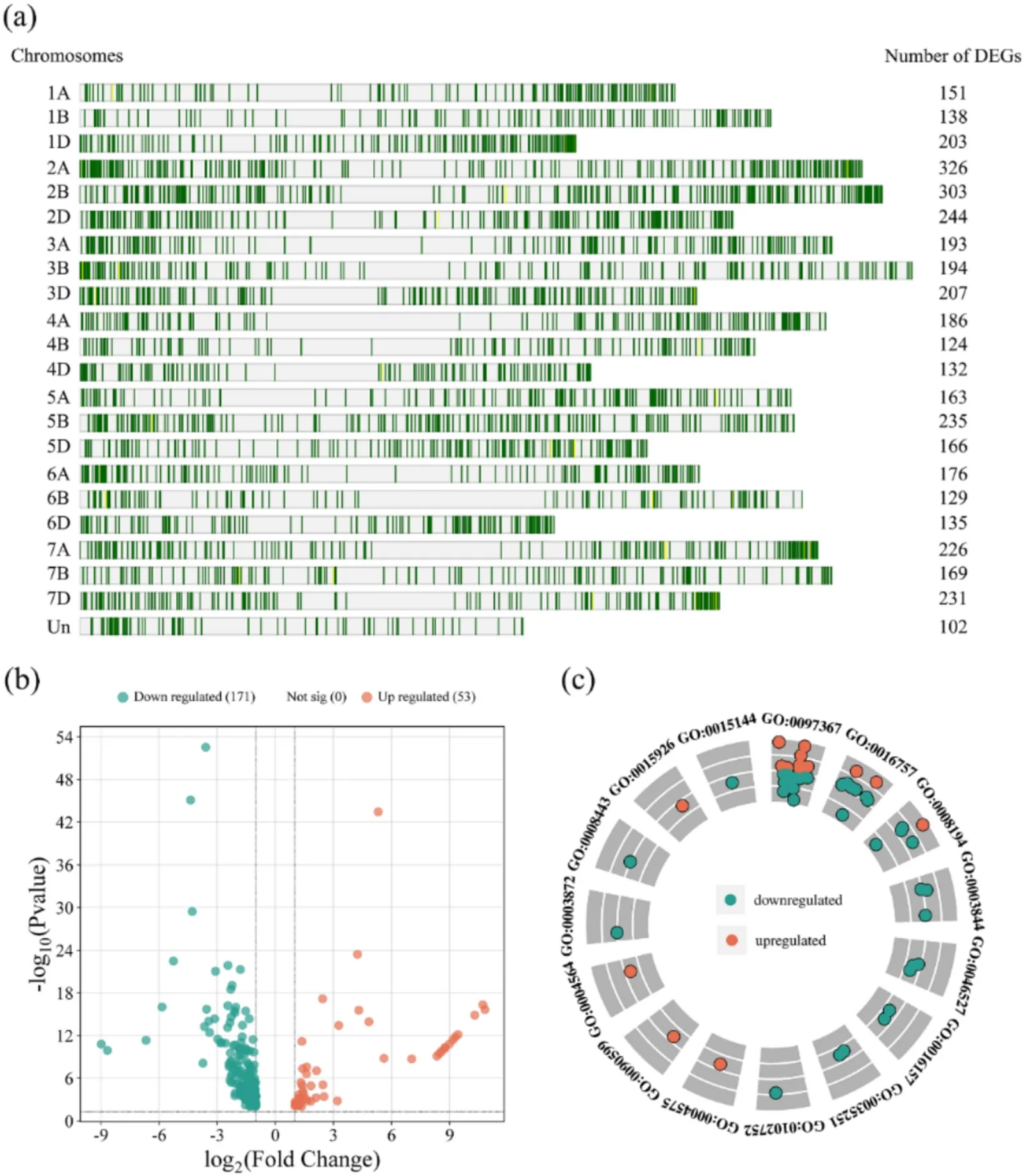
Distribution of DEGs across wheat chromosomes. **a** DEGs between AC6012 and AC6361 on 21 wheat chromosomes. **b** DEGs on chromosome 7A. **c** GO Terms associated with carbohydrate metabolism involving DEGs on Chromosome 7A

There were 5737 DEGs between AC6012 and AC6361, with AC6012 as the reference background. The distribution of DEGs on 21 wheat chromosomes was shown by transcriptome analysis (Fig. 5a). Two hundred and twenty-six DEGs were detected on chromosome 7A, among which 172 genes were downregulated and 54 were upregulated in AC6361 relative to AC6012 (Fig. 5b). Gene ontology (GO) analysis of 226 DEGs revealed that 33 DEGs were enriched in 15 GO terms related to carbohydrate metabolism (Fig. 5c).

### Validation and clone of *TaGLK-A1*

**Fig. 6:**
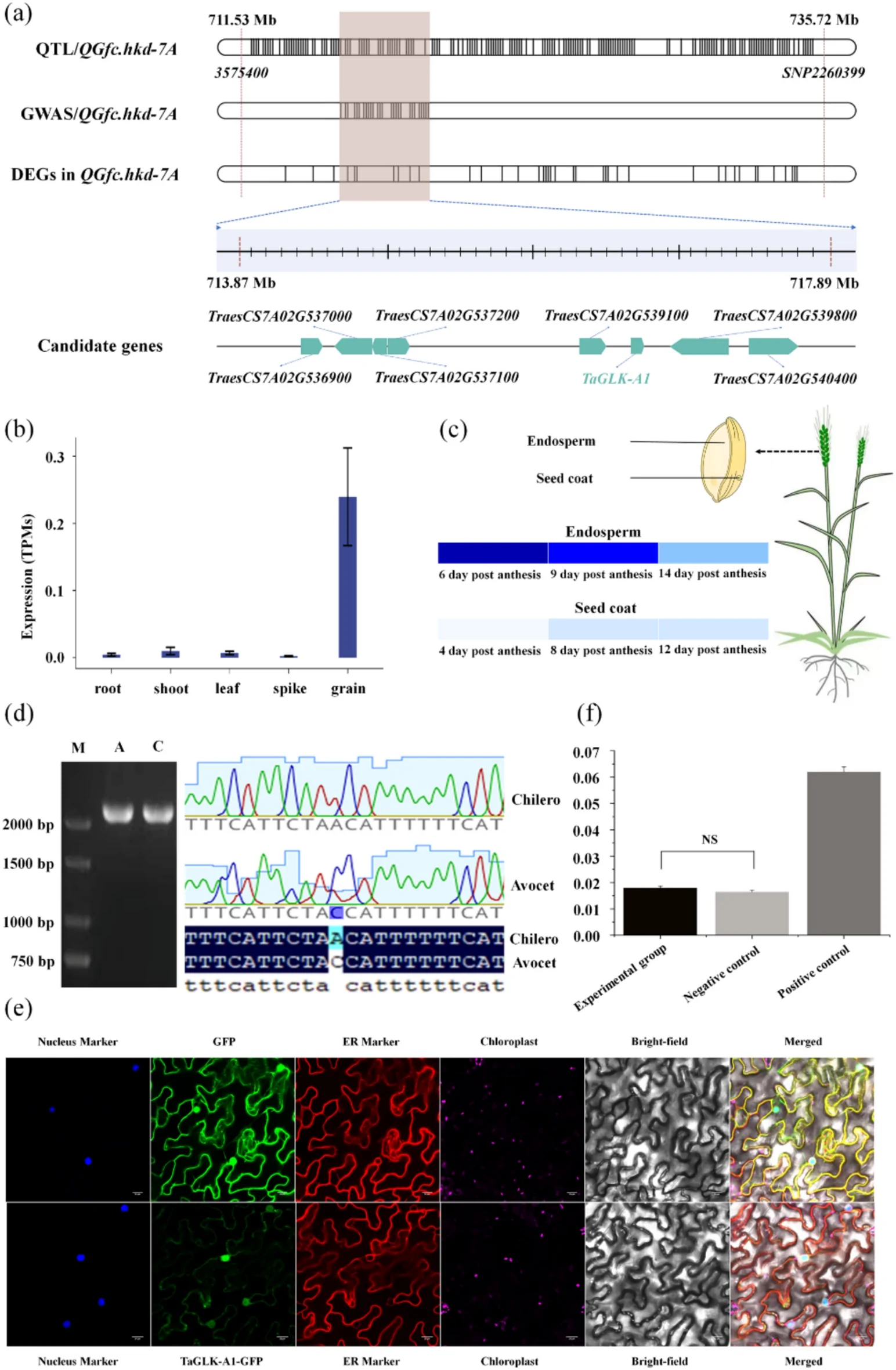
Screening, cloning and characterization of *TaGLK-A1*. **a** Eight candidate genes identified by GWAS, linkage mapping and transcriptome analysis. **b** Gene expression of *TaGLK-A1* across tissues. **c** The expression of *TaGLK-A1* at different developmental stages; TPM, transcripts per million. **d** Cloning of *TaGLK-A1* in Avocet and Chilero, M, Marker; A, Avocet; C, Chilero. **e** Subcellular localization of *TaGLK-A1* using tobacco leaves. **f** Transactivation activity analysis of *TaGLK-A1*. Experimental group: the effector construct pGreenII-62sk-GAL4-TaGLK-A1; Negative control: the construct pGreenII-62sk-GAL4; Positive control: the construct pGreenII-62sk-GAL4-VP16. NS indicates no significant difference

*TaGLK-A1* (*TraesCS7A02G539600*), which is functionally associated with carbohydrate metabolism, was identified as a strong potential candidate gene within the *QGfc.hkd-7A* interval. Transcriptome analysis revealed 8 DEGs among the 47 high-confidence genes in this interval (Fig. 6a). Through the combination of GWAS, QTL mapping, transcriptome data and public database annotations (http://wheat.cau.edu.cn/wGRN; https://www.ebi.ac.uk/gxa/home) (Chen et al. 2023; Gillies et al. 2012), *TaGLK-A1* was prioritized as the most promising candidate (Fig. 6b-c). Furthermore, the expression level of *TaGLK-A1* in AC6361 was lower than that in AC6012, and this gene was enriched in the GO:0015144 term (Fig. 5c).

Full-length amplification primers were designed based on the nucleotide sequence specificity of *TaGLK-A1* between two genotypes (Table 2), which showed a single clear band of approximately 1500 bp in Avocet and Chilero (Fig. 6d). Sequencing results showed that the full-length sequences of *TaGLK-A1* in both varieties were 1545 bp, consisting of four exons and three introns, which encoded a 338 amino acid protein. A missense mutation was identified at position + 109 bp (C → A) in the coding region, resulting in an amino acid substitution from proline (Pro) to threonine (Thr) (Fig. 6d).

**Table 2:** Primer sequences for amplification of *TaGLK-A1*

Primer	Primer sequences (5′–3′)
*TaGLK-A1-F*	AAGCCAAGGGTGGTATGGACAGT
*TaGLK-A1-R*	ATCGCGTGCATAGGTTGGGCTAC

Subcellular localization assays revealed that the GFP-tagged TaGLK-A1 protein co-localized with the nuclear marker BZR1, indicating that *TaGLK-A1* is localized to the nucleus (Fig. 6e). Transactivation activity assays revealed that the experimental group (pGreenII-62sk-GAL4-TaGLK-A1) showed no significant difference compared to the negative control (pGreenII-62sk-GAL4), whereas the positive control (pGreenII-62sk-GAL4-VP16) exhibited strong transactivation activity, indicating that *TaGLK-A1* lacks transactivation activity (Fig. 6f).

## Discussion

### Combination analysis of *QGfc.hkd-7A* for fructan content via multi-omics

Recently, the genetic dissection of complex quantitative traits has been improved through the combined application of GWAS, QTL mapping and transcriptome sequencing (Guo et al. 2019; Zhao et al. 2023). This convergent analytical strategy has emerged as a highly effective way to identify candidate genes in crops, providing greater resolution than any of these techniques alone (Yang et al. 2023; Zhao et al. 2021). In this study, we employed such a multi-pronged genomic pipeline to pinpoint *QGfc.hkd-7A*, a stable QTL on chromosome 7AL governing wheat grain fructan content. Through the convergent use of GWAS, QTL mapping and transcriptome analysis, we successfully delimited the interval to 4.02 Mb, encompassing eight DEGs. It should be noted that the transcriptome analysis was conducted using only two lines (AC6012 and AC6361) selected solely based on their extreme and stable phenotypic differences across multiple environments, following an extreme phenotype screening strategy commonly used in wheat transcriptome studies (Shi et al. 2024; Wu et al. 2025; Yuan et al. 2023). The cross-validation across multiple environments (*H*^*2*^ = 0.77) demonstrates that our multi-omics approach not only enhances mapping resolution but also effectively distinguishes stable loci from environment-specific signals, offering a robust framework for cloning genes underlying complex metabolic traits.

### Identification of candidate genes for*TaGLK-A1*

Previous studies have shown that fructan accumulation occurs mainly during the first phase of grain development, known as the grain enlargement phase, which lasts two weeks post-anthesis (Pepler et al. 2006; Shewry et al. 2012; Verspreet et al. 2013). During this developmental stage, the acid invertases 1-SST and 6-SFT exhibit high levels of enzymatic activity. These enzymes may actively hydrolyze sucrose and thereby reduce sucrose concentrations and promote fructan synthesis in developing wheat grains while controlling osmotic flow into the developing grain (Verspreet et al. 2013). In this study, we utilized the publicly accessible Expression Atlas database (https://www.ebi.ac.uk/gxa/home) to assess eight potential DEGs. The results indicated that only *TaGLK-A1* (*TraesCS7A02G539600*) demonstrated expression patterns consistent with those of fructan biosynthetic enzymes during the initial stages of grain development. However, *TaGLK-A1* does not encode a known fructan metabolic enzyme, suggesting that its influence on fructan accumulation may be indirect, potentially through modulating substrate transport or metabolic signaling.

Indeed, fructan metabolism is tightly coupled to sucrose transport and signaling. It has been reported that fructosyltransferase enzymes evolved from acid or vacuolar invertase enzymes through a few mutational changes, and fructosyltransferase and invertase enzymes have similar functions and a high degree of amino acid similarity (Chatterton and Harrison 1997; Veenstra et al. 2017; Vijn and Smeekens 1999). Some fructan exohydrolases and cell wall invertases are closely related (Coninck et al. 2005; Van den Ende et al. 2004; Van den Ende et al. 2002). Although the fructan metabolism pathway has not yet been fully elucidated, sucrose is known to play an important role, and protein kinases and type 2A protein phosphatases are also important components in the sucrose signal transduction pathway to induce fructan synthesis (Martínez-Noël et al. 2006, 2009). *TaGLK-A1* (*TraesCS7A02G539600*) is annotated with the molecular function “carbohydrate transmembrane transporter activity” (GO:0015144), whose child terms predominantly encompass transmembrane transporter activities, including sugar transmembrane transporter, D-glucarate transmembrane transporter and protein–phosphocysteine–sugar phosphotransferase activities. Collectively, these annotations position *TaGLK-A1* within the carbohydrate metabolic network that supports fructan biosynthesis.

While the convergent evidence from genomic position, differential expression and sequence variation supports the prioritization of *TaGLK-A1* as the primary candidate, it is important to acknowledge that this designation rests on correlational rather than causal evidence. Although integrative genomic approaches frequently identify promising candidates within QTL intervals, the true causal relationship can only be established through subsequent functional validation such as gene silencing, overexpression or genome editing (Lin et al. 2024). In wheat, CRISPR-Cas9-mediated knockout or knock-in has been increasingly employed to confirm the causal role of candidate genes identified through mapping approaches (Waites et al. 2025; Kan et al. 2023). Direct evidence demonstrating that *TaGLK-A1* modulates fructan content in planta remains to be established and will be the focus of our future research.

Despite these limitations, the cumulative evidence—including its physical location within the *QGfc.hkd-7A* interval, its consistent differential expression between high- and low-fructan lines—collectively positions *TaGLK-A1* as the strongest candidate gene for wheat grain fructan content. Consequently, we have proceeded with its molecular cloning and functional characterization to explore its potential regulatory role in wheat fructan metabolism.

### Cloning and subcellular localization of *TaGLK-A1*

Previous research has effectively isolated and functionally characterized key fructan metabolic enzymes in wheat, such as 1-SST, 1-FFT and 6-SFT (Kawakami and Yoshida 2002, 2005; Yoshida et al. 2004; Gao et al. 2009; Yue et al. 2011; Wang et al. 2018), demonstrating their involvement in fructan polymerization and stress tolerance through heterologous expression and transgenic validation (Bie 2011; He 2016). However, these studies have predominantly concentrated on structural genes, leaving the upstream transcriptional regulators responsible for directing carbon partitioning into the fructan biosynthetic pathway during grain development largely unexplored. In this study, we successfully cloned *TaGLK-A1* (*TraesCS7A02G539600*), a G2-like transcription factor family member carrying a Pro109Thr substitution. Notably, the temporal expression pattern of *TaGLK-A1* during the early stages of grain development exhibits a notable correlation with that of fructan biosynthetic enzymes, suggesting a regulatory role rather than a catalytic one. This observation represents a paradigm shift from traditional enzymatic characterization toward an emphasis on transcriptional regulation, thereby identifying *TaGLK-A1* as a critical regulator in the metabolism of grain fructans.

The nuclear compartmentalization of transcription factors is essential for their regulatory roles, as demonstrated by *TaDOF6* (Ding et al. 2025), *TaNAC47* (Zhang et al. 2016), *OsNF-YB1* (Liu et al. 2025) and *OsMADS14* (Feng et al. 2022), which are exclusively localized to the nucleus to activate genes involved in carbohydrate metabolism during grain development. In alignment with its classification as a G2-like transcription factor, *TaGLK-A1* (*TraesCS7A02G539600*) was observed to reside exclusively within the nucleus in our subcellular localization assays, precisely co-localizing with the BZR1 nuclear marker. This nuclear localization situates *TaGLK-A1* at the core of the fructan metabolic network, facilitating the direct regulation of biosynthetic enzyme expression and linking genetic mapping with the mechanistic regulation of grain fructan accumulation.

The lack of transactivation activity in *TaGLK-A1* is not an isolated phenomenon among functional wheat transcription factors. Comparable findings have been documented for several wheat transcription factors, including the heat shock factor *TaHsfC3-4* (Ma et al. 2024), C2H2-type zinc finger proteins TaZFP23 (Ye and Tang 2025) and TaZFP8-5B (Huang et al. 2024), as well as various members of the TaBES1 family (Zhang et al. 2025). These proteins, despite their absence of intrinsic transcriptional activation capacity, are integral to stress responses and developmental processes. The absence of self-activation in *TaGLK-A1* implies that it may need interacting partners or specific post-translational modifications to fulfill its regulatory role. This observation aligns with the emerging paradigm that transcriptional regulation frequently relies on protein complexes.

## Conclusions

In the present study, through the combination of genome-wide association study, linkage mapping and transcriptome analysis, we identified and cloned a novel strong candidate gene, *TaGLK-A1*, a G2-like transcription factor localized in the nucleus, which is associated with fructan content in wheat grain.

## Supplementary Information

Below is the link to the electronic supplementary material.

Supplementary file1 (XLSX 1411 KB)

Supplementary file2 (XLSX 855 KB)

## Data Availability

The genotype data, phenotype data and mapping files (.xlsx) supporting the QTL analysis are provided as Supplementary_Data_1_LinkageMapping_ICIMapping. The RNA-seq data are provided as Supplementary_Data_2_RNAseq_Expression_Matrix.
